# Light-Triggered and Sustained Delivery of Dexamethasone Using Chitosan-Coated PLGA Nanoparticles for Posterior Eye Disease Treatment

**DOI:** 10.1021/acsomega.6c02332

**Published:** 2026-06-26

**Authors:** Lorenzo Guidi, Maria Grazia Cascone, Gaia Riccio, Sofia Patri, Rupali Dabas, Lorenzo Lavista, Andrea Camposeo, Dario Pisignano, Elisabetta Rosellini, Nazila Kamaly

**Affiliations:** † Department of Civil and Industrial Engineering, 9310 University of Pisa, Pisa 56122, Italy; ‡ Department of Chemistry, 4615 Imperial College London, London SW7 2AZ, U.K.; § Istituto NanoscienzeCNR, Pisa I-56127, Italy

## Abstract

Degenerative eye disorders (DEDs) such as age-related macular degeneration and diabetic retinopathy pose significant therapeutic challenges due to anatomical barriers limiting drug access to posterior ocular tissues. Current treatments, including intravitreal injections, offer localized delivery but require repeated administration, increasing risks and reducing patient compliance. This study introduces a dual-functional nanoparticulate platform for sustained and light-triggered delivery of dexamethasone, a corticosteroid widely used in DED management. Poly­(lactic-*co*-glycolic acid) (PLGA) nanoparticles were fabricated via nanoprecipitation, coloaded with dexamethasone and the near-infrared (NIR)-responsive dye IR820, and coated with chitosan to enhance mucoadhesion. Physicochemical characterization confirmed nanoscale size, monodispersity, and positive surface charge upon chitosan coating. Drug release studies revealed biphasic kinetics, with chitosan-coated formulations exhibiting prolonged release compared to uncoated particles. Under irradiation (800 nm), photothermal activation mediated by a NIR-absorbing dye induced a 2.0–2.3-fold increase in dexamethasone release during early exposure phases, enabling on-demand dosing. Cyto- compatibility assays on NIH-3T3 fibroblasts confirmed high cell viability (>70%) across all formulations and exposure conditions. These findings demonstrate a robust, light-responsive, mucoadhesive nanoparticle system with the potential to improve therapeutic precision and reduce invasiveness in posterior eye disease treatment.

## Graphical abstract

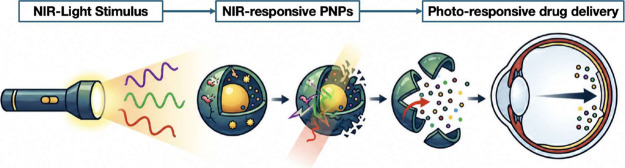

## 1 Introduction

Degenerative eye disorders (DEDs) such as age-related macular degeneration and diabetic retinopathy represent a growing public health concern, contributing significantly to the global burden of blindness and visual impairment. As of 2020, more than 2.2 billion people worldwide were affected by some form of visual deficit, with over 200 million suffering from moderate to severe loss.^1–6^ While the human visual system plays a dominant role in sensory information processing, therapeutic access to posterior eye tissues remains highly constrained by the unique anatomical and physiological barriers of the eye. Both static components—such as the vitreous body—and dynamic systems like aqueous humor flow restrict the penetration and bioavailability of administered drugs, limiting their effectiveness in treating deep retinal pathologies.^7–9^

Traditional ophthalmic treatments, including topical drops, systemic therapies, and intraocular injections, often fail to achieve sustained therapeutic levels within the posterior segment.^10,11^ Among them, intravitreal injection has become the clinical gold standard for delivering drugs directly to retinal tissues due to its ability to bypass many ocular barriers.^10,12–17^ However, this invasive method is not without drawbacks: repeated administration is frequently required to maintain drug concentrations, leading to decreased patient compliance and an elevated risk of complications such as intraocular inflammation, infection, retinal detachment, and increased intraocular pressure. Implantable systems offer a degree of relief through prolonged release profiles, but they still require surgical intervention for placement or removal and often lack spatial precision or dosing flexibility.^1,12–14,17–19^

These limitations have fuelled increasing interest in the design of advanced drug delivery systems (DDSs) that can enhance treatment efficacy while improving patient experience. Among these, polymeric nanoparticles (PNPs) have emerged as a particularly versatile class of carriers.^5,20,21^ Engineered from biocompatible and biodegradable materials, PNPs offer a unique platform for controlled drug release, enhanced solubility and stability of encapsulated agents, and minimized systemic toxicity. Capable of transporting molecules ranging from small drugs to proteins and nucleic acids, they hold strong potential for overcoming the barriers that hinder conventional ocular therapies.^21–24^ To circumvent these severe physiological bottlenecks, there is an intensifying research trajectory focused on the engineering of smart, stimulus-responsive drug delivery systems (DDSs). These advanced architectures are uniquely capable of enhancing localized therapeutic efficacy, providing sustained release profiles, and significantly improving the overall patient compliance experience. Recent comprehensive evaluations have highlighted how tailoring matrix polymer degradation, interfacial properties, and stimulus-triggered kinetics can radically bypass conventional barriers, paving the way for highly translatable nanomedicine frameworks.^25–27^

Sustained-release delivery systems are especially vital in chronic posterior eye conditions, where prolonged exposure to therapeutics can slow or prevent disease progression. Nanoparticles with mucoadhesive coatings, such as chitosan, are particularly well-suited for this application, as they can adhere to ocular mucosal surfaces and resist rapid clearance. Poly­(lactic-*co*-glycolic acid) (PLGA), a widely studied biodegradable polymer, allows for gradual drug release while breaking down into nontoxic metabolites—supporting long-acting delivery and reducing the burden of frequent administration.^24,28–31^ These advanced surfaces can actively form noncovalent bonds with the sialic acid residues of the ocular mucosal matrix, thereby drastically extending localized retention times and resisting rapid physiological clearance mechanisms.^32–34^

More recently, innovations in smart nanotechnology have enabled the development of stimuli-responsive carriers that adjust drug release rates in response to environmental triggers such as pH, temperature, enzymatic activity, or light. These precision-tuned systems open new avenues for tailored, on-demand therapies, while light-responsive nanocarriers hold great promise in ocular applications due to the eye’s natural transparency.^28–31,35–43^

Near-infrared (NIR) light, which offers deep tissue penetration with minimal absorption by endogenous chromophores such as blood and melanin, can be leveraged to activate photothermal drug release mechanisms within nanoparticles. These systems convert absorbed light into heat, triggering the release of encapsulated compounds from thermosensitive matrices. Several photothermal agents—including gold nanoparticles, conductive polymers like PDOPA, and dyes such as indocyanine green (ICG) or IR820—have demonstrated effective heat generation and drug release under safe irradiation parameters.^20,41,44–47^

To date, several near-infrared (NIR)-responsive nanoparticulate systems have been explored for ocular applications, frequently utilizing inorganic photothermal agents or complex chemical conjugation strategies to tether stimuli-responsive moieties.^5,15^ While promising, many of these existing frameworks suffer from critical shortcomings, including potential long-term ocular toxicity of heavy metal cores, intricate synthetic pathways that hinder translational scalability, and a tendency to display rapid clearance due to a lack of surface-engineered ocular retention mechanisms.^10,16^ Furthermore, many light-triggered ocular systems lack a reliable mechanism to balance long-term sustained baseline delivery with acute on-demand dosing. To address these gaps, this study introduces a distinct, fully organic, dual-functional nanoarchitecture. By combining a biodegradable poly­(lactic-*co*-glycolic acid) (PLGA) core physically coloaded with dexamethasone and the clinically viable dye IR820 via a simple, single-step nanoprecipitation track, this platform circumvents complex chemical synthesis while maintaining sharp photothermal responsiveness.^48,49^ Crucially, the integration of a nontoxic, cationic chitosan shell uniquely adds a diffusional barrier to suppress initial burst leakage and ensure prolonged baseline retention, establishing a robust system capable of switching between long-term sustained release and precise, light-triggered dosing windows.^13,50^

In this study, we report on a PLGA-based nanoparticulate platform developed via nanoprecipitation and functionalized with a chitosan shell to enhance the mucoadhesion and biocompatibility. The system is dual-loaded with dexamethasone (DEX) and the FDA-approved NIR-absorbing dye IR820, enabling both sustained release and photothermal-triggered delivery upon external light stimulation. Dexamethasone, a potent anti-inflammatory corticosteroid commonly used in treating DEDs such as AMD, often suffers from poor posterior penetration and systemic side effects when delivered by conventional.^48,49,51^ By leveraging a light-activated delivery approach, this platform aims to provide a responsive, localized, and minimally invasive treatment alternative. Comprehensive evaluation includes an analysis of physicochemical properties, passive and NIR-stimulated release behavior, and in vitro cytocompatibility.^17^

## 2 Materials and Methods

### 2.1 Materials and Reagents

Resomer RG 504 H—Poly­(D,l-lactide-*co*-glycolide) (L:G 50:50, Mw 36–54k), dexamethasone (DEX) (392.46 Da), chitosan (Chit) (low Mw: 50–190k Da, deacetylation 75–85%), IR820 (849.47 Da), Tween 20, acetonitrile (ACN), dimethylformamide (DMF), ethanol, acetic acid, phosphate buffered saline (PBS), Ultra 15 Amicon Filters (MWCO 100k), Minisart Syringe filters (0.45 μm) were purchased by Sigma-Aldrich. Phosphate buffer pH 7.0 (PBS), trypsin, fetal bovine serum (FBS), penicillin, streptomycin, l-glutamine, and DMEM medium were purchased from Merck Italy. Murine fibroblasts of the NIH-3T3 line were supplied from the Unit of Cellular, Molecular, and Developmental Biology (University of Pisa).

### 2.2 Nanoparticle Preparation

PLGA nanoparticles (PLGA-NPs) were prepared using the nanoprecipitation technique.^52^ Briefly, a stock solution of PLGA 20 mg/mL was prepared in ACN, which was then stored at −20 °C. The organic phase was obtained by withdrawing 720 μL of stock and adding 280 μL of ACN. The aqueous phase consisted of 10 mL of ultrapure water. The organic phase was slowly added dropwise to the aqueous phase under magnetic agitation (1000 rpm). The reaction was allowed to proceed for 4 h to achieve complete evaporation of the organic solvent. The solution was then filtered through 0.45 μm cellulose filters into Ultra 15 Amicon Filters (MWCO 100 kDa) and centrifuged at 3000*g* for 20 min (Thermo Scientific—Thermo sorvall ST8R) at 4 °C. The nanoparticles were subsequently suspended in a total of 7 mL of ultrapure water to achieve a concentration of 0.4 mg/mL. To maximize nanoparticle recovery and minimize nonspecific adsorption during ultrafiltration, Amicon Ultra filters equipped with low-binding regenerated cellulose membranes were utilized. Following filtration and subsequent freeze-drying, the total mass output of the recovered solid nanoparticle cake was empirically quantified using an analytical balance to a precise decimal place (0.01 mg). This direct gravimetric confirmation consistently verified optimal formulation recovery and demonstrated negligible mass loss due to membrane fouling or structural entrapment.

PLGA-DEX and PLGA-DEX-IR820 nanoparticles were prepared by a nanoprecipitation technique, where DEX and IR820 were physically encapsulated within the PLGA matrix rather than chemically conjugated to the polymer. Nanoparticles were obtained by modifying only the composition of the organic phase and the organic solvent system, while the aqueous phase and mixing/dripping parameters were kept constant across all formulations. Specifically, PLGA, DEX, and IR820 were codissolved in an organic phase consisting of acetonitrile (ACN) diluted 9:1 (v/v) with dimethylformamide (DMF) to enhance the solubility of the hydrophobic components and promote drug encapsulation efficiency.^53^

For the preparation of PLGA nanoparticles, PLGA (2880 μg) was withdrawn from a stock solution prepared by dissolving 20 mg of polymer in 5 mL of solvent. For PLGA-DEX nanoparticles, the organic phase consisted of PLGA (2880 μg) and dexamethasone (500 μg) withdrawn from their respective stock solutions, where dexamethasone was prepared at 17% (w/v) by dissolving 2 mg in 1 mL of solvent. For PLGA-DEX-IR820 nanoparticles, PLGA (2880 μg), dexamethasone (500 μg), and IR820 (600 μg) were combined, with IR820 prepared at 21% (w/v) by dissolving 20 mg in 1 mL of solvent. For drug-loaded formulations, the organic solvent system consisted of ACN supplemented with DMF at a 9:1 (v/v) ratio, while for unloaded PLGA nanoparticles, acetonitrile alone was used, and IR820 was dissolved in DMF alone. All other formulation and processing parameters were kept constant.

Each formulation was subsequently coated with Chitosan. The procedure involved dripping the NP suspension (2:1 v:v) obtained after the first centrifuge in a Chitosan solution under mild agitation (150 rpm) overnight. Three different concentrations of Chitosan (0.01, 0.1, 0.3% w/v) were dissolved in ultrapure water at pH 3.5 with the addition of 0.5 M NaCl.^54,55^ The NP-Chitosan suspensions were then filtered through 0.45 μm cellulose filters into Ultra 15 Amicon Filters (MWCO 100 kDa) and centrifuged at 3000*g* for 20 min at 4 °C,^56^ thus obtaining PLGA/CHT, PLGA-DEX/CHT, and PLGA-DEX-IR820/CHT.

Finally, all formulations were freeze-dried (Telstar–Cryodos) for further testing and storage. All NP suspensions were quick-frozen through dry ice after the addition of 10% w/v sucrose and freeze-dried at −55 °C and 0.047 mbar for 24 h.^57^

### 2.3 Characterization Techniques

#### 2.3.1 Size, PDI, and Zeta Potential (DLS)

The hydrodynamic diameter and polydispersity index (PDI) of the nanoparticles were measured using Dynamic Light Scattering (DLS) on a Malvern Zetasizer Ultra. For analysis, 20 μL of the centrifuged nanoparticle suspension was diluted in 1 mL of ultrapure water or PBS, where specified. Measurements were carried out in triplicate at room temperature, using disposable cuvettes, a fixed scattering angle of 90°, and an attenuator setting of 8. The zeta potential of all formulations was evaluated via Electrophoretic Light Scattering (ELS), also using the Malvern Zetasizer Ultra. Sample preparation followed the same procedure as above, and measurements were taken in triplicate at room temperature under an applied voltage of 150 V.

The stability at 37 °C of the final formulation (PLGA-DEX-IR820/CHT) was evaluated through suspending it at a concentration of 3 mg/mL in PBS 5 mM + 0.05%Tween 20 for a week. The same formulation was also tested for post-freeze-drying stability through reconstitution in water by analyzing the Z average and PDI before and after a cycle of freeze-drying with the addition of 10% sucrose as a cryoprotectant.

To evaluate the success and entity of the chitosan coating, Z-average, PDI, and zeta potential were also evaluated in comparison to the w/v percentage of chitosan in the coating solution.

#### 2.3.2 Morphology by TEM

The morphology of the NPs was examined using TEM (JEOL 2100Plus TEM) with an electron accelerating voltage (AV) of 200 kV.

#### 2.3.3 FTIR (ATR) for Drug–Polymer Interaction Confirmation

Fourier-transform infrared spectroscopy with attenuated total reflectance (FTIR-ATR) was used to analyze the lyophilized nanoparticles (Tensor 27 FTIR spectrometer, Bruker) to confirm successful drug encapsulation and the presence of a chitosan surface coating. A small portion of the freeze-dried sample was carefully applied to the ATR crystal to enhance spectral clarity. Spectra were recorded across the 400–4000 cm^–1^ range to identify characteristic functional groups and evaluate potential interactions among the polymer, drug, and chitosan.

#### 2.3.4 UV–Vis Spectrophotometry

DEX and IR820 content in the nanoparticles were quantified by UV–vis spectrophotometry. The filtrates collected from the centrifuge tubes equipped with Amicon filters were freeze-dried for 48 h. After drying, each sample was reconstituted in 1 mL of deionized water for UV–vis analysis. Standard solutions of the Dexamethasone and IR820 were prepared by dissolving DEX and IR820 in water at the following concentrations: 0.0167, 0.0200, 0.0250, 0.0333, 0.0500 mg/mL. The UV absorbance of both the filtrates and standards was measured at 255 nm (Dexamethasone) and 833 nm (IR820) using a Nanodrop spectrophotometer. All measurements were performed in triplicate. Drug loading was evaluated by measuring the presence of both in the aqueous phase, filtered through the Amicon filters, in terms of Loading Efficiency (LE) and Encapsulation Efficiency (EE), calculated according to the following equations (eqs 1 and 2):

$$\mathrm{LE}\;(\%)=\frac{m_{\mathrm{in}}-m_{\mathrm{lost}}}{m_{\mathrm{NPs}}}\times 100$$ 1

$$\mathrm{EE}\;(\%)=\frac{m_{\mathrm{in}}-m_{\mathrm{lost}}}{m_{\mathrm{drug}}}\times 100$$ 2

where *m*_in_ is the employed drug mass for the preparation of the nanoparticles, *m*_lost_ is the UV/vis quantified drug lost during its encapsulation, and *m*_NPs_ is the total output mass of nanoparticles produced.

#### 2.3.5 In Vitro Release Studies

Nanoparticle (NP) samples were prepared at a concentration of 3 mg/mL in PBS 5 mM + 0.05 v/v % Tween20 and incubated in triplicate at 37 °C in Eppendorf tubes. A low ionic strength was selected to preserve the electrostatic stabilization of chitosan-coated PLGA nanoparticles, limiting double-layer compression and salt-induced aggregation. Buffer capacity was sufficient under the periodic medium-refresh schedule adopted.^58^ At predetermined time points (1, 2, 4, 8, 24, 48, 96, 168, and 336 h), the NPs were collected and transferred to AmiconUltra-0.5 centrifugal filters (10 kDa MWCO), then centrifuged at 14000×*g* for 4 min at 37 °C. The retained NPs were resuspended in fresh PBS and returned to incubation until the next sampling time point. The released drug was quantified by analyzing the Amicon-filtered nanoparticle suspension through UV/vis analysis at 255 nm. Standard solutions of dexamethasone were prepared by dissolving DEX and IR820 in PBS 5 mM + 0.05%Tween20 at the following concentrations: 0.0167, 0.0200, 0.0250, 0.0333, 0.0500 mg/mL. The filtrates (and standards) were analyzed using a Nanodrop UV–vis spectrophotometer by measuring absorbance at 255 nm in order to quantify the amount of present drug in the Amicon-filtered nanoparticle suspension medium. To eliminate potential optical interference from polymer fragments, surfactant micelles, or residual nanoparticle scattering during spectrophotometric quantification, all UV–vis measurements were standardly baseline-corrected against a blank matrix consisting of the exact release buffer (5 mM PBS with surfactant) subjected to identical centrifugal filtration processing.

#### 2.3.6 Kinetic Modeling

To better understand the drug release mechanism, experimental data were fitted to commonly used mathematical models describing drug release kinetics. Linear fitting was performed to identify the predominant release mechanism. The following models (eqs 3–7) were evaluated:^59–62^

- Zero Order (constant release rate, typically applicable in nondegradable release systems):
$$Q_{t}=Q_{0}+K_{0}\cdot t$$ 3
- First Order (release rate proportional to remaining drug):
$$\ln(Q_{r})=\ln(Q_{0})-K_{1}\cdot t$$ 4
- Hixson–Crowell (Release rate limited mainly by degradation of the carrier matrix, typically applicable in systems subject to degradation):
$$Q_{0}^{1/3}-Q_{t}^{1/3}=K_{a}\cdot t$$ 5
- Higuchi (Release limited mainly by diffusion through the matrix, usually applied for nondegradable systems):
$$Q_{t}=K_{H}\cdot \sqrt{t}$$ 6
- Korsmeyer–Peppas (Semiempirical model, used to describe release mechanisms where several phenomena coexist):
$$\frac{M_{t}}{M_{\infty }}=K_{p}\cdot t^{n}$$ 7

In these equations: *Q*_*t*_ and *M*_*t*_ represent the cumulative amount of drug released at time *t*, *Q*_0_ is the initial amount of drug released (often assumed to be zero), *Q*_*r*_ is the amount of drug remaining at time *t*, *M*_∞_ is the total amount of drug initially loaded into the nanoparticles, *K*_0_, *K*_1_, *K*_*a*_, *K*_*H*_, and *K*_*p*_ are the rate constraints for the respective models and *n* is the release exponent indicating the release mechanism in the Korsmeyer–Peppas model. Model fitting was performed by linearizing the equations and evaluating the correlation coefficient (*R* ^2^) for each model to determine the best fit.

#### 2.3.7 Light-Triggered Release Study

To assess the influence of photothermal effects on the drug release kinetics, an in vitro release protocol was implemented by using the setup schematized in Figure 1a. Specifically, 6 mg of PLGA-DEX-IR820 nanoparticles were suspended in 2 mL of release medium. The suspension was enclosed in a cellulose dialysis membrane (MWCO 12000 Da) and placed in 40 mL of release medium maintained at 37 °C with continuous stirring. Drug release was monitored across three consecutive ON/OFF laser irradiation cycles, as illustrated in Figure 1b. Aliquots of 3 mL were taken from the external medium every 10 min and replaced with fresh buffer to maintain sink conditions. The concentration of released DEX was quantified by UV–vis spectroscopy, with absorbance measured at 255 nm. For irradiation a 800 nm Ti:sapphire laser (Mira, Coherent), pumped by a Verdi V10 laser (Coherent), was used. Laser irradiation was performed for a duration of 8 min with 950 mW impinging onto the sample. This specific exposure time frame was chosen based on preliminary thermalization assays indicating that photothermal temperature stabilization occurs within this window, balancing dosing efficiency with safety parameters designed to minimize prolonged intraocular laser exposure. The beam exhibited a Gaussian spatial profile and an elliptical shape, characterized by semiaxes of 13 and 7.5 mm, respectively. The resulting beam area is about 3 cm^2^. This yields an average irradiance of about 0.31 W/cm^2^.

**Figure 1:**
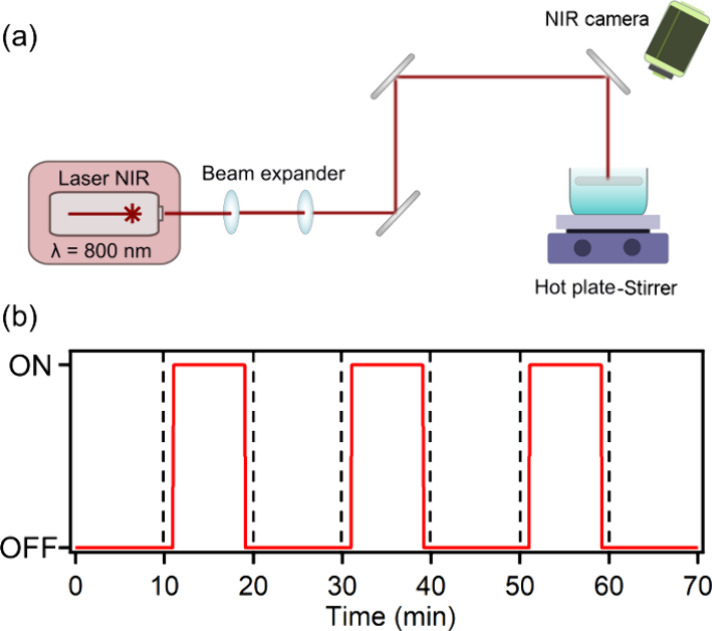
(a) Schematics of the experimental setup used for light-triggered release. (b) Illustration of the phases of the light-triggered release experiments. The red lines highlight the time intervals in which the NIR laser is switched ON, whereas the dashed vertical lines highlight the time points at which a 3 mL aliquot of the solution was taken for subsequent spectroscopic analysis.

#### 2.3.8 Cell Culture Studies

3T3 fibroblast cells were cultured in 75 cm^2^ flasks using DMEM supplemented with 10% fetal bovine serum (FBS), 2% l-glutamine, and appropriate concentrations of penicillin and streptomycin (5000 U/mL and 5 μg/mL, respectively). Cultures were maintained at 37 °C in a humidified atmosphere containing 5% CO_2_. When cells reached 70–80% confluency, they were harvested by trypsinization and used for cytotoxicity assays.

#### 2.3.9 Analysis of In Vitro Cytotoxicity

Cell viability was first assessed using an XTT colorimetric assay following exposure to nanoparticle extracts. Extracts were prepared by dispersing various nanoparticle formulations (PLGA, PLGA/CHT, PLGA-DEX, PLGA-DEX/CHT, PLGA-DEX-IR820, and PLGA-DEX-IR820/CHT) in PBS at a concentration of 100 μg/mL. The suspensions were incubated at 37 °C for 5 days. After incubation, the supernatants were collected, sterilized via 200 nm cellulose membrane filtration, and applied to cells.

3T3 cells were seeded in 96-well plates at a density of 4 × 10^4^ cells/mL (150 μL per well). After 24 h of cell attachment, 100 μL of each extract was added to the wells in sextuplicate. Positive controls, hereon named C+ (medium consisting in 10% v/v Triton X-100 in PBS) and negative controls, hereon named C– (PBS alone), were included, each in six replicates. Following a 24-h exposure period, 50 μL of XTT reagent was added to each well, and the plates were incubated for an additional 4 h at 37 °C in the dark. Absorbance was recorded at 430 nm with a reference wavelength of 650 nm using a microplate reader (Biorad, United States). Cell viability was calculated relative to the negative control.

A second assay was conducted to evaluate cell viability after direct exposure to the nanoparticle suspensions. Only formulations containing both dexamethasone and IR820, with and without chitosan coating (PLGA-DEX-IR820 and PLGA-DEX-IR820/CHT), were tested. Nanoparticles were sterilized by UV irradiation for 30 min and resuspended in PBS at three concentrations: 25, 50, and 100 μg/mL.

Cells were seeded in 96-well plates at a density of 4 × 10^4^ cells/mL for the 24-h assay and 2 × 10^4^ cells/mL for the 72-h assay (150 μL per well). After incubation, cells were treated with the nanoparticle suspensions. For each formulation and concentration, six replicates were included, along with six wells each for positive and negative controls. The rest of the protocol—including XTT addition, incubation, and absorbance measurement—followed the same procedure as in the first assay.

## 3 Results and Discussion

### 3.1 Particle Size, Morphology, and Surface Properties

TEM imaging (Figure 2) confirmed that all nanoparticle variants possessed a uniform, spherical shape with smooth exterior surfaces. According to DLS data (Suppl. Mat. Table S1), the PLGA nanoparticles had an average hydrodynamic diameter (Zav) of 90.9 ± 1.8 nm and a PDI of 0.13 ± 0.02, indicating a small and consistent size distribution. The addition of dexamethasone and IR820 did not significantly alter the size, with PLGA-DEX and PLGA-DEX-IR820 nanoparticles measuring 96.5 ± 0.6 and 100.1 ± 5.7 nm, respectively, and maintaining similarly low PDI values (0.17 ± 0.02 and 0.19 ± 0.04). These findings highlight the nanoscale dimensions and monodispersity of the uncoated particles. The step-by-step chemical assembly, intermediate nanostructures, and the final core–shell architecture of the dual-functional platform are schematically illustrated in Figure 3, along with a detailed depiction of the noncovalent and electrostatic interfacial interactions governing matrix cohesion.

**Figure 2:**
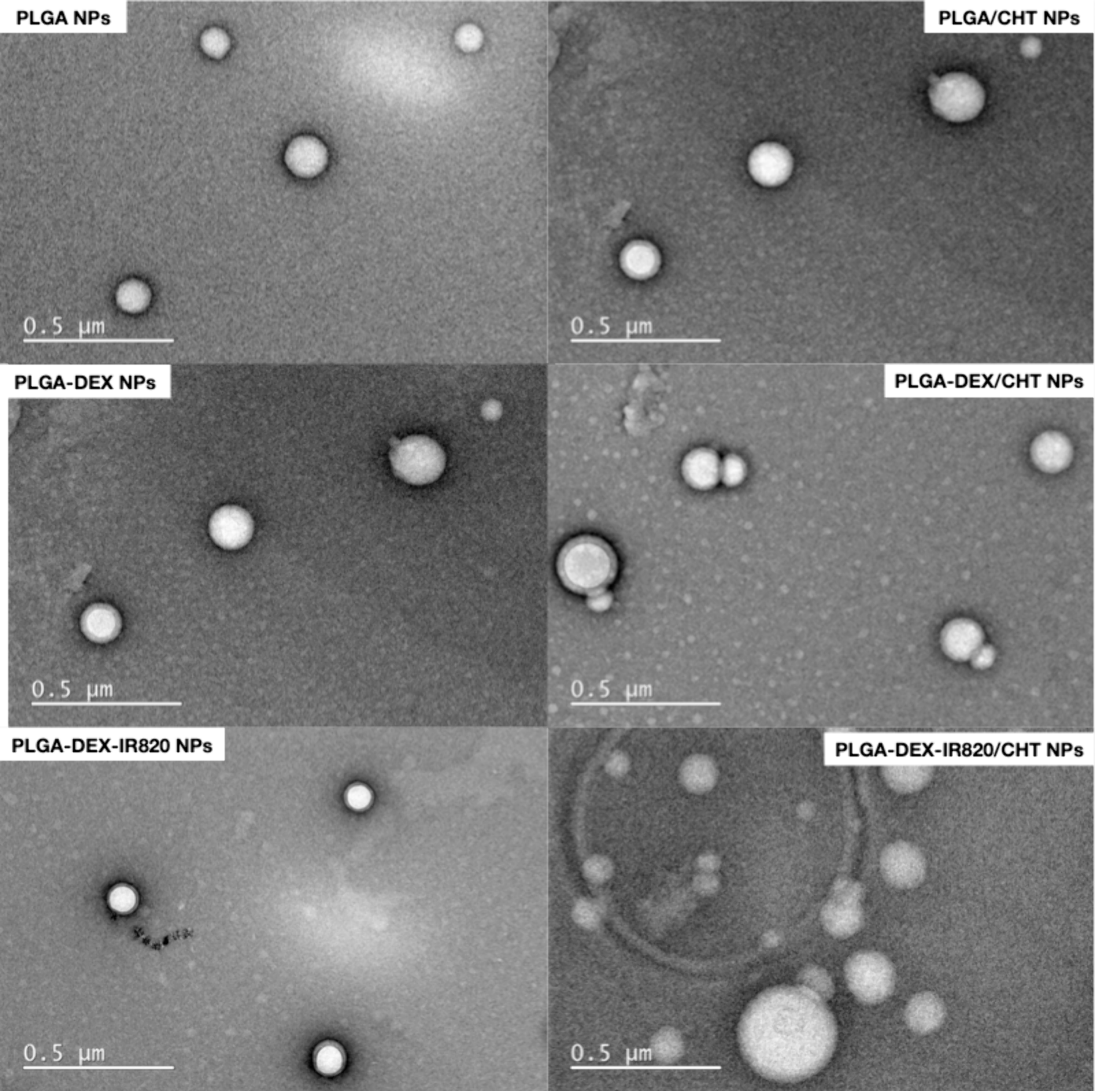
TEM imaging results for all nanoprecipitation-based formulations

**Figure 3:**
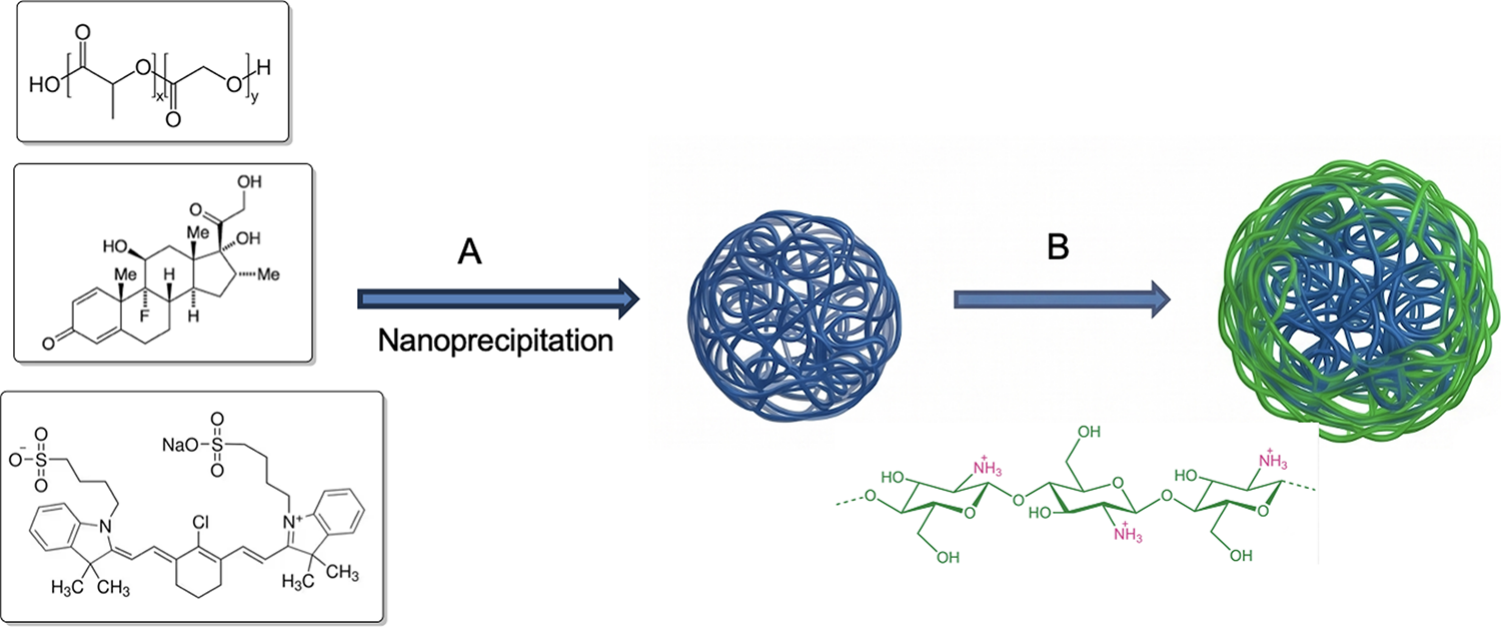
Schematic representation of the formulation assembly and matrix interactions. (A) Single-step nanoprecipitation of PLGA, dexamethasone, and IR820 yields a hydrophobic intermediate core with a net negative surface charge. (B) Subsequent electrostatic interfacial assembly with a cationic chitosan polymer layer at pH 3.5 to produce the final mucoadhesive nanocarrier.

Introduction of a chitosan coating markedly increased particle size across all chitosan-modified formulations (PLGA-CHT, PLGA-DEX/CHT, and PLGA-DEX-IR820/CHT), with Z-average values rising to a range of 450–500 nm, attributed to the formation of an outer chitosan shell. Furthermore, neither the suspension of PLGA-DEX-IR820/CHT NPs in 0.05 M PBS + 0.05% Tween 20 (Zav change −5.2%, ζ-potential change −5.7%) nor the reconstitution of the nanoparticles post-freeze-drying significantly impacted their dimensions or PDI (Zav change −12.7%, ζ-potential change +10.2%).

Zeta potential measurements further distinguished coated from uncoated particles: while uncoated NPs exhibited negative surface charges between −23 and −12 mV—consistent with PLGA’s negative charge—chitosan-coated variants showed a positive charge, ranging from +32 to +42 mV. This reversal in surface charge, furthermore evident from Figure 4 which depicts the variation of *Z* average, PDI, and ζ-potential with respect to the concentration of chitosan in the coating solution, confirms successful deposition of the cationic chitosan layer and indicates the robustness of the coating process.

**Figure 4:**
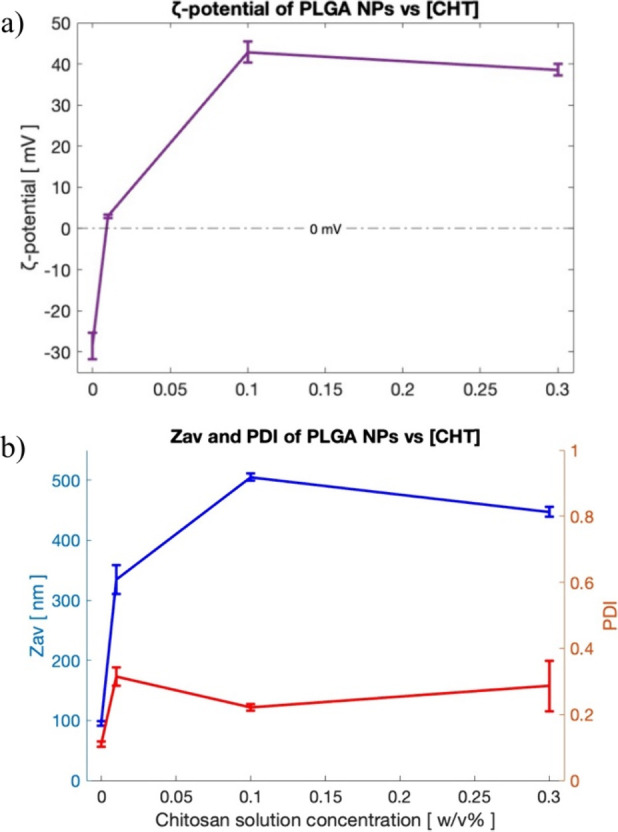
(a) Variation of the zeta potential (above), (b) *Z*-average and PDI (below) of coated PLGA NPs with respect to different concentrations of dissolved chitosan in the coating solutions (0.01, 0.1, 0.3%).

### 3.2 FTIR Spectral Analysis

Figure 5 reports the FTIR spectra of different nanoparticle formulations. The dexamethasone (DEX) spectrum exhibits distinct peaks: 1662 cm^–1^, attributed to carboxyl −C=O stretching vibration, and at 892 cm^–1^ (C–F group axial deformation).^63,64^ The successful encapsulation of DEX in both PLGA-DEX NPs and PLGA-DEX-IR820 NPs is confirmed by the presence of characteristic peaks corresponding to both PLGA and DEX in their respective spectra.

**Figure 5:**
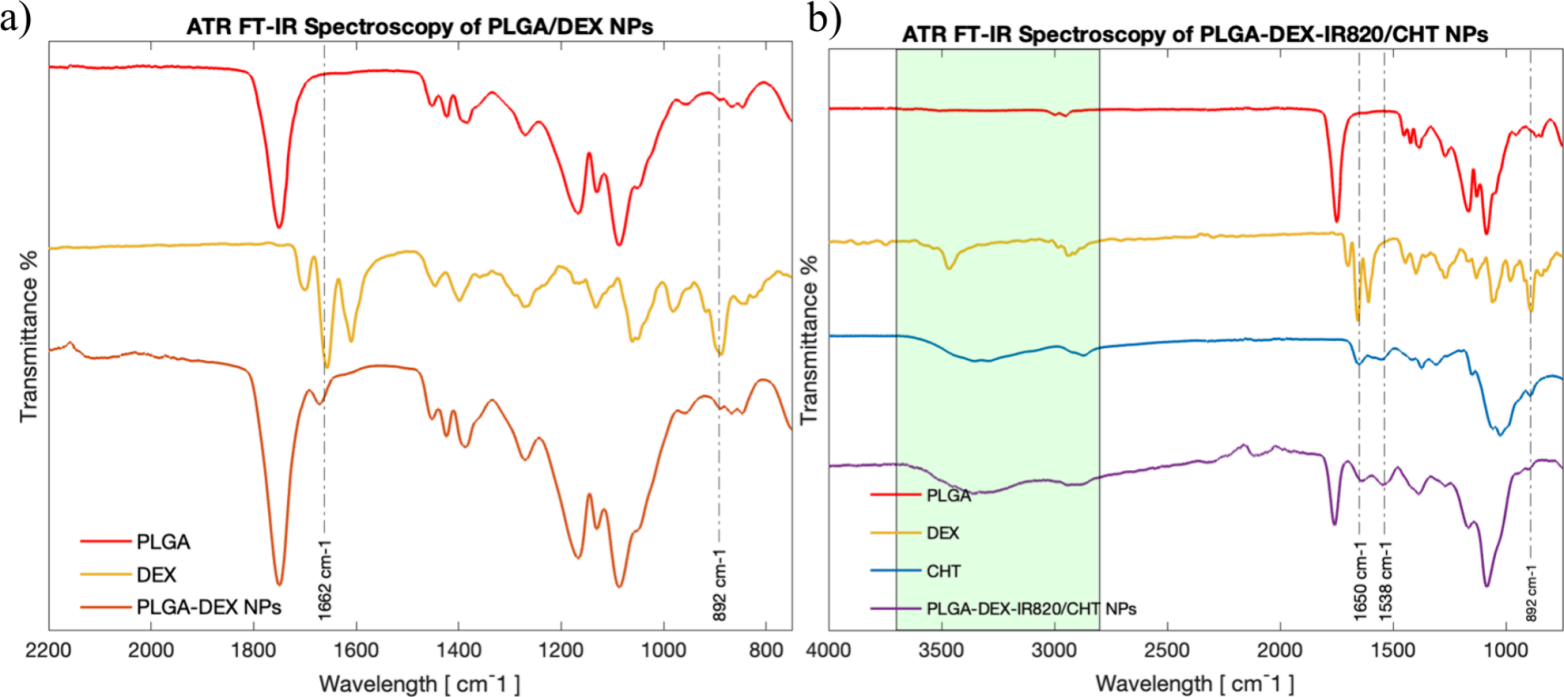
FTIR-ATR spectra of: (a) PLGA NPs, dexamethasone, and PLGA-DEX NPs; (b) PLGA NPs, Dexamethasone, Chitosan and PLGA-DEX-IR820/CHT NPs.

The FTIR spectrum of chitosan shows a broad absorption band at 3400 cm^–1^, ascribed to stretching vibration of −NH2 and −OH groups,^50,65^ along with a peak at 1652 cm^–1^ attributed to the amide group.^50,66^ These chitosan-related features are also evident in the spectrum of PLGA-DEX-IR820/CHT NPs, together with the characteristic peaks of PLGA and DEX, thereby confirming the presence of the chitosan coating in the final formulation. Moreover, the presence of a slight shift and attenuation of the 3400 cm^–1^ band of the chitosan, as well as the attenuation of characteristic DEX peaks, could suggest the presence of hydrogen bonds or noncovalent interactions between the drug and the chitosan layer.^67,68^

The combination of macroscopic surface charge inversion and molecular-level infrared spectral shifts provides complementary, scientifically sound confirmation of successful shell deposition. While direct microscopic visualization of soft macromolecular coatings via standard high-vacuum TEM can induce localized polymeric collapse or artifacts, the distinct ∼ 50 mV zeta potential transition combined with the characteristic amide and hydroxyl FTIR modifications firmly validates the interfacial assembly of a cohesive, stable chitosan layer around the hydrophobic PLGA cores.

### 3.3 UV–Visible Spectrophotometry

The calibration curve for DEX and IR820 in water was validated by calculating the correlation coefficient, showing an *R* ^2^ value of 0.99. This allowed for the estimation of drug concentration from sample absorbance using the following linear equations (eqs 8 and 9):

$$A_{\mathrm{DEX}}=29.82\cdot c_{\mathrm{DEX}}-0.11$$ 8

$$A_{\mathrm{IR}820}=33\cdot c_{\mathrm{IR}820}+0.16$$ 9

In eqs 8 and 9, the intercept values represent the matrix-specific background baseline factors, which account for minor residual instrument noise, scattering, and sample matrix reflections. All spectrophotometric absorbance measurements were performed using a NanoDrop spectrophotometer operating at an automated, microvolume optimized optical path length of 1 mm, allowing for direct concentration calculations via the Beer–Lambert law. UV–visible spectrophotometric analysis confirmed the successful encapsulation of DEX within the PLGA-DEX NPs, with a loading efficiency (LE) of 7.6 ± 0.5% and an encapsulation efficiency (EE) of 47.6 ± 3.3%. The LE and EE values for all tested formulations are reported in *Suppl. Mat.* Table S2.

Co-encapsulation of the IR820 dye increased drug loading, possibly due to the presence of a cosolvent which accelerated DEX precipitation,^53^ while for both chitosan-coated nanoparticles (PLGA-DEX/CHT NPs and PLGA-DEX-IR820/CHT NPs), a slight reduction in both drug LE (−7%) and dye LE (−12.7%) was observed. This decrease may be attributed to the prolonged suspension of the loaded nanoparticles in an acidic environment and the need for an additional centrifugation cycle, increasing their loss.

#### 3.3.1 Drug Release Profiles

Drug release analysis revealed a release profile (% DEX released vs time [h]) consistent with that typically observed for nanoparticulate drug delivery systems. To quantify the released drug, a calibration curve for DEX in PBS-Tween80 solution was first developed and validated, yielding an *R* ^2^ value of 0.99 with the following linear equation (eq 10):

A=20.62·c+0.06 10

The release profile was evaluated for both PLGA-DEX NPs and PLGA-DEX/Chit NPs, and in both cases exhibited a biphasic behavior (Supporting Information). Mat. Figure S1), characterized by an initial burst release followed by a slower and sustained release phase, successively reaching a plateau. Both formulations demonstrated controlled release properties, with cumulative DEX release reaching approximately 18% for PLGA-DEX NPs and 8% for PLGA-DEX/Chit NPs after 336 h. The strongly reduced release observed in chitosan-coated nanoparticles was expected and is hypothesized to stem from the additional polymeric layer grafted onto the PLGA core, which hinders drug diffusion toward the release medium.^50,69,70^

To further elucidate the underlying release mechanisms, the initial burst release phase was subjected to linear fitting by using various mathematical models. As summarized in *Supporting Information*, Mat. Table S3, the Korsmeyer-Peppas model provided the best fit for PLGA-DEX NPs, with an *R* ^2^ value of 0.95, an *n* value of 0.47, and a *K*_H_ value of 1.16, while the Higuchi model proved to be the best fit for PLGA-DEX/CHT NPs, with an *R* ^2^ value of 0.99 and *a K*_H_ value of 0.24 (*Supporting Information*). Mat. Figure S2) The Korsmeyer-Peppas model is widely used to analyze drug release from polymeric systems. A good fit to this model indicates that the release follows anomalous (non-Fickian) transport, where both diffusion and polymer relaxation or erosion contribute to the release kinetics. The value of the release exponent (*n*) provides insight into the dominant mechanism, with values between 0.45 and 0.89 typically suggesting a combination of Fickian diffusion and polymer matrix swelling or degradation.^71^ The Higuchi model is commonly applied to systems in which drug release occurs predominantly via Fickian diffusion. The strong correlation between experimental data and this model suggests that diffusion through the polymer matrix is the primary release mechanism during the early stages, while polymer erosion or degradation likely contributes to the sustained release observed in the later phases.^72^ The stark divergence in the mathematical fitting models between the two formulations reflects a fundamental shift in the physical transport mechanisms governing the drug release. Upon electrostatic deposition of the hydrophilic shell, the PLGA-DEX/CHT nanoparticles transition to a near-perfect Higuchi model fit (*R* ^2^ = 0.99). Here, the dense, highly hydrated chitosan layer acts as a stable stationary boundary. This shell dampens the immediate impact of core surface erosion, shifting the rate-limiting step entirely to pure, Fickian diffusion through the tortuous channels of the swelling core–shell matrix.

The cumulative passive drug release profiles observed over 336 h—ranging from approximately 8% for the coated assemblies to 18% for the raw cores—demonstrate a highly controlled, sustained release trajectory. This low fractional release rate highlights the performance of the formulation as a long-acting intraocular depot. By minimizing early burst leakage and maintaining a highly conservative passive diffusion baseline, the nanocarrier ensures a prolonged therapeutic lifespan capable of providing sustained baseline dosing over multimonth timelines, while preserving a significant drug fraction for active, light-triggered demand cycles.

### 3.4 Photothermal Response

Temperature data recorded through an IR camera shows a distinct and reproducible temperature increase in correspondence with the irradiation with the NIR laser (Figure 6). Upon immersion of the sample (at room temperature) in the PBS medium (at 37 °C), a variation of the sample temperature was observed in the first 10 min due to thermalization. Upon irradiation with the NIR laser, a 7.6 °C increase of the sample temperature in coincidence with the irradiated area is observed (Figure 6). Dye activation was also confirmed through visual inspection of the bleached sample following three cycles of NIR irradiation, with respect to the unirradiated sample (Suppl. Mat. Figure S3). The temperature profiles recorded via infrared thermography confirm the high specificity of the photothermal effect. As illustrated in Figure 6d, the control baseline consists of irradiated PLGA-DEX nanoparticles lacking the IR820 dye (blue line), which exhibits a completely flat thermal profile. This is compared against both nonirradiated PLGA-DEX-IR820 nanoparticles (green line) and the experimental group of irradiated PLGA-DEX-IR820 nanoparticles (red line).

**Figure 6:**
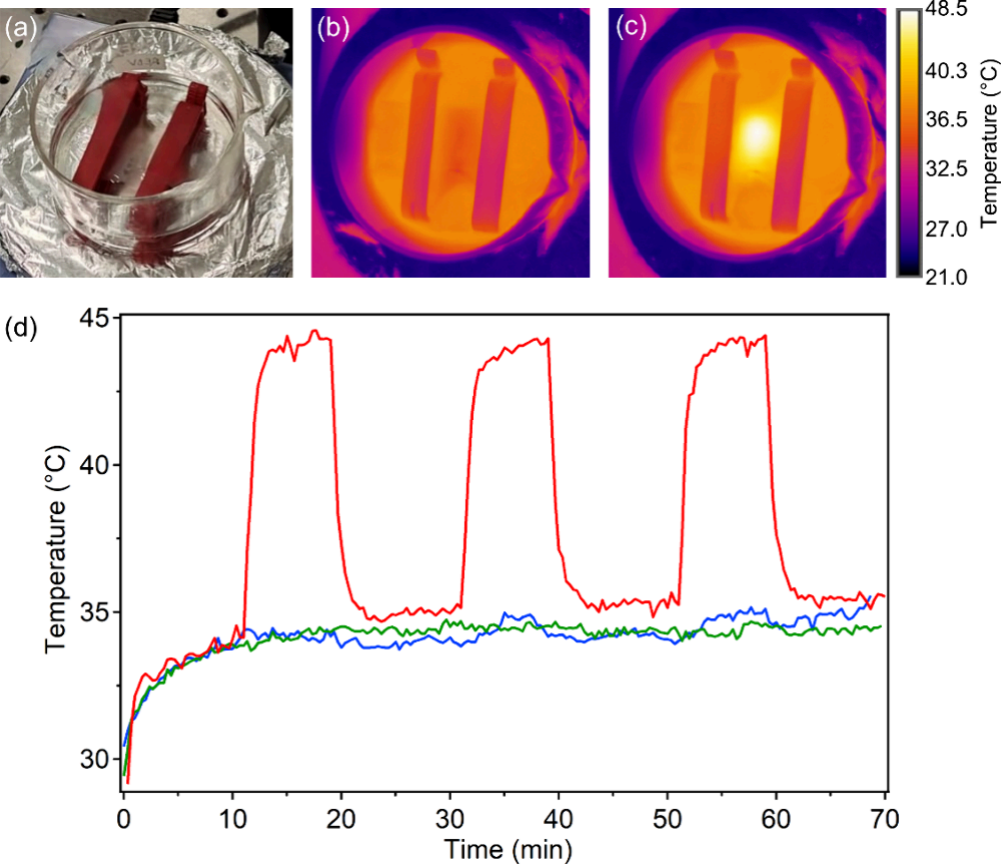
(a) photograph of the sample. (b,c) Thermal images of the sample before (b) and during (c) the NIR irradiation. (d) Temperature increase of irradiated PLGA-DEX-IR820 NPs (red line) with respect to PLGA-DEX NPs (blue line) and nonirradiated PLGA-DEX-IR820 NPs (green line) following three cycles of 800 nm NIR irradiation.

The release profiles indicate that the effect of the NIR irradiation intervals (red) was time-dependent (Figure 7). In the early stage (0–40 min), the combined average of the PLGA-DEX-IR NPs data sets exhibited a marked enhancement of release compared with baseline, with NIR intervals yielding increments approximately 2.0–2.3-fold greater than adjacent white intervals. The baseline average (PLGA-DEX NPs) remained essentially flat across all intervals, thereby confirming that the enhancement was specific to the experimental samples. At a late stage (40–70 min), release increments were minimal, and no significant differences between NIR-irradiated and nonirradiated intervals were detected (red/white ratio ≈ 0.9) (Figure 8).

**Figure 7:**
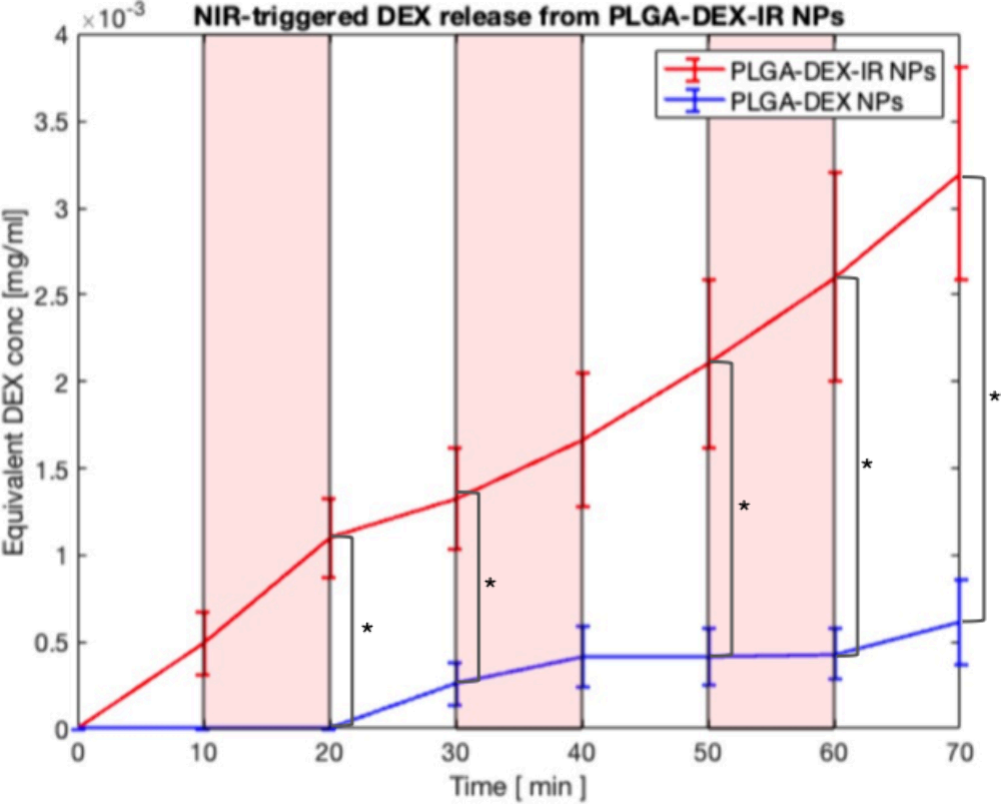
NIR-triggered release from PLGA-DEX and PLGA-DEX-IR (short for PLGA-DEX-IR820) NPs given a NIR source at 800 nm and an average irradiance of 310 mW/cm^2^, applied to the NP suspension during red intervals highlighted in red. Data are expressed as Mean ± SD (*n* = 3). Statistical analysis was performed using an independent Student’s *t* test at each individual time point. * Indicates a statistically significant difference (*P* < 0.05) in cumulative drug release between the NIR-irradiated PLGA-DEX-IR NPs and the nonirradiated PLGA-DEX control group.

**Figure 8:**
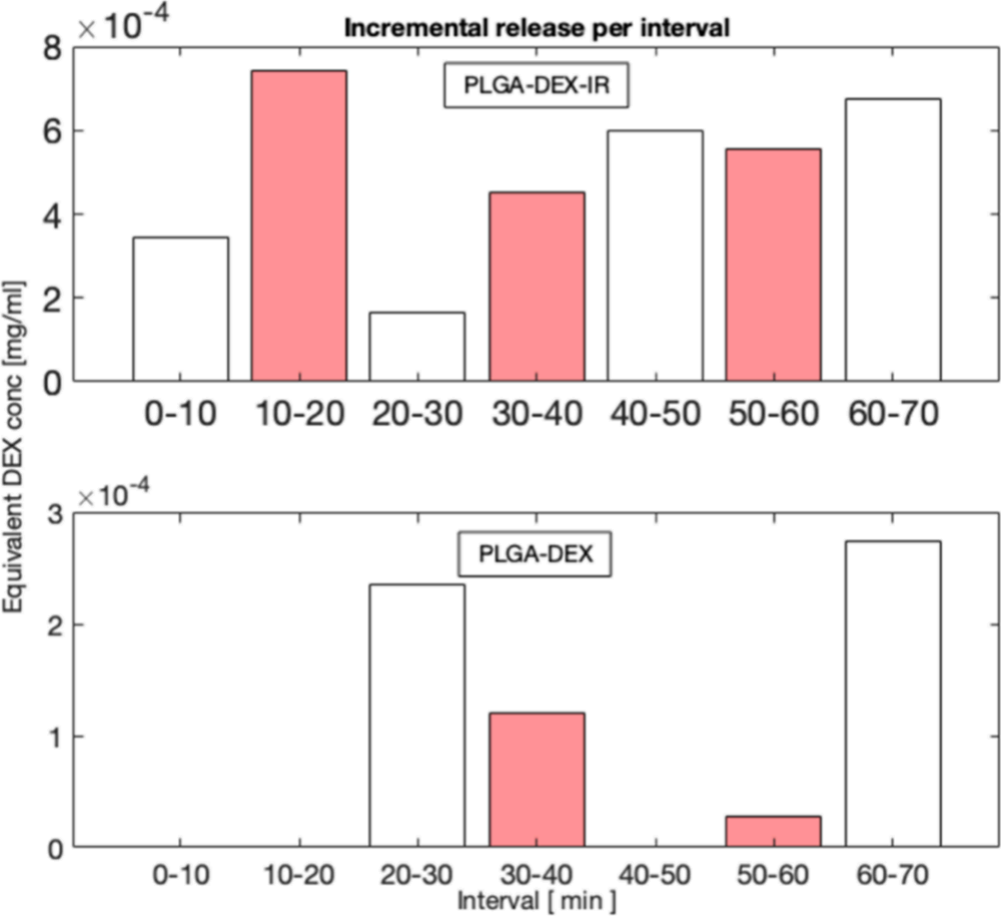
NIR-triggered release from PLGA-DEX and PLGA-DEX-IR (short for PLGA-DEX-IR820) NPs. Each column represents the drug-release ratio (variation between adjacent intervals).

Given the very small absolute changes during this time frame, these late-phase variations are more likely attributable to a variation of the release kinetics with a gradual relative reduction favoring sustained release, while NIR-absorbing properties remain stable. The interval analysis graph and bar plots further illustrate that the NIR-induced enhancement was confined to the early phase, gradually diminishing as the system approached equilibrium.

### 3.5 Cytocompatibility

Ensuring cytocompatibility is a fundamental requirement for any nanoparticle-based drug delivery system intended for ocular applications. To assess the safety of the developed formulations, the cytotoxicity of the nanoparticles was evaluated on 3T3 fibroblasts using an XTT assay.

In the first test, cells were exposed to extracts obtained from five different NP formulations. As shown in Figure 9, all formulations—except for the positive control (C+) and the coated formulations (loaded and unloaded with only DEX)—maintained high cell viability, with no statistically significant differences compared to the negative control (C−) (α = 0.01). These results suggest that, while most of the formulations elicited no cytotoxic effect on 3T3 cells, the chitosan coating of the two mentioned formulations (PLGA/CHT and PLGA-DEX/CHT) seems to have had a slight influence on vitality, which nonetheless remained over 70%.

**Figure 9:**
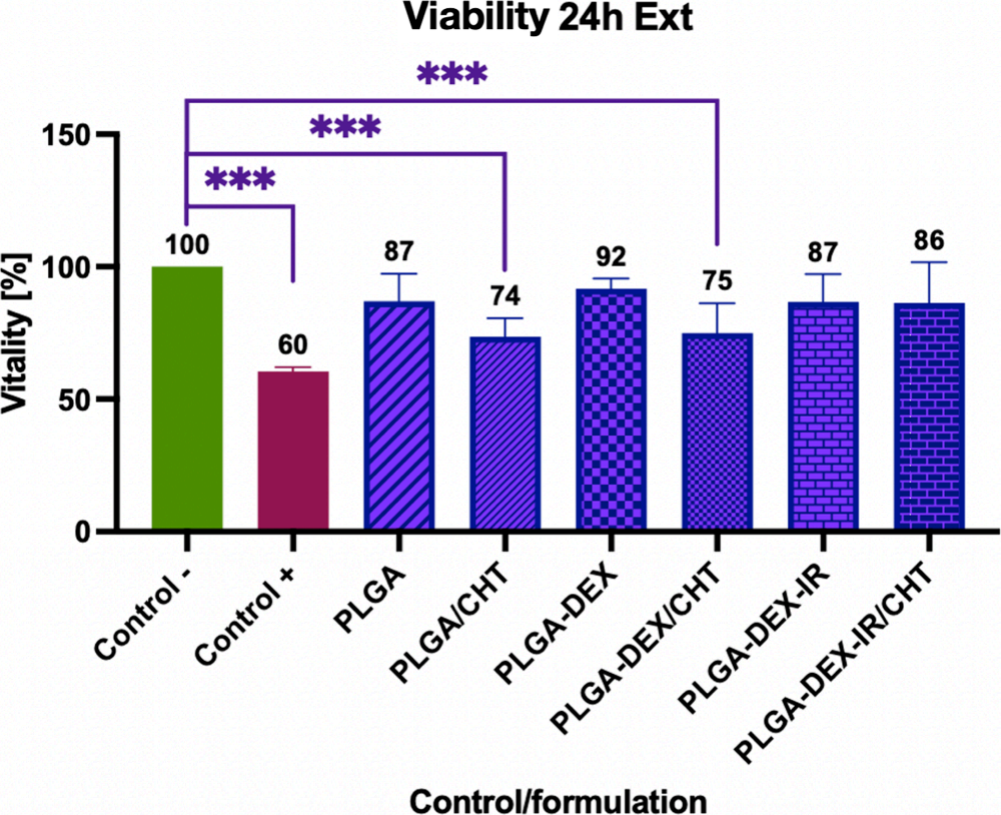
Cytocompatibility of extracts from all formulations with respect to the negative (PBS) and positive (10% v/v Triton X-100 in PBS) control samples. IR is short for IR820 in the mentioned formulations.

This prompted a second test to assess the cytotoxicity of the final formulations (PLGA-DEX-IR820 and PLGA-DEX-IR820/CHT) to ascertain whether direct contact with our final product (coated or uncoated) might trigger any adverse reaction from cells. In the second test, the cytotoxicity was evaluated at increasing concentrations (25, 50, and 100 μg/mL) of PDI (PLGA-DEX-IR820 NPs) and PDIC (PLGA-DEX-IR820/CHT NPs) for both 24 and 72 h. As reported in Figure 10, no significant reduction in cell viability was observed under any condition. Moreover, there was no indication of a dose-dependent effect, and the two incubation times yielded comparable results. This is consistent with the well-established biocompatibility of PLGA and Chitosan, both widely used in drug delivery systems,^73–75^ and with previous reports of low cytotoxicity for IR820.^47,76^

**Figure 10:**
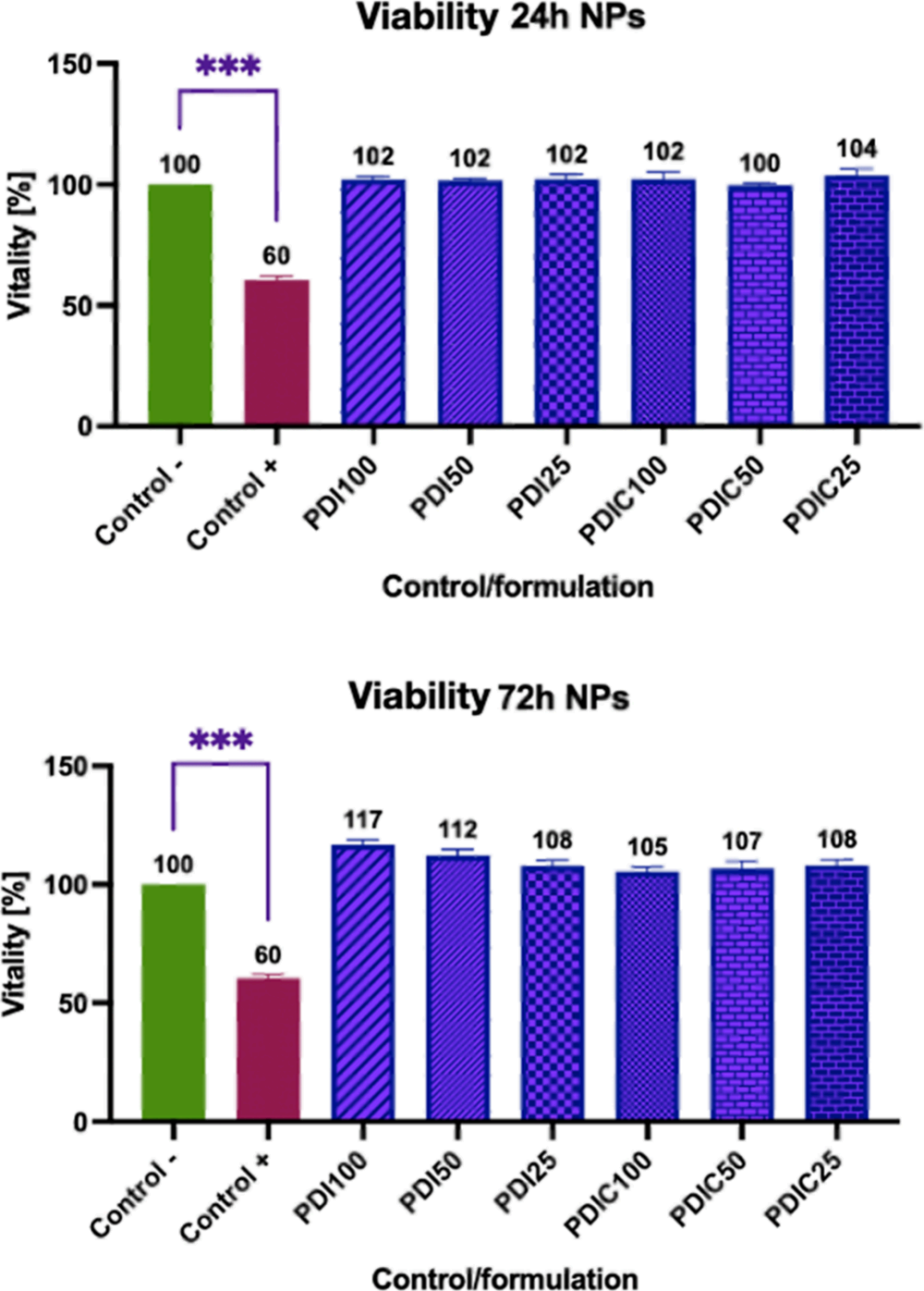
Cytocompatibility of suspensions from selected formulations at three different concentrations with respect to the negative (PBS) and positive (10% v/v Triton X-100 in PBS) control samples. Viability was evaluated and plotted at 24 (above) and 72 h (below). PDI is short for PLGA-DEX-IR820 NPs and PDIC is short for PLGA-DEX-IR820/CHT NPs.

The extract cytotoxicity assays presented in Figure 9 were conducted utilizing an absolute nanoparticle concentration of 100 μg/mL across all evaluated formulations. To ensure a rigorous and controlled toxicological assessment, a single, unified master batch of both the PLGA-DEX-IR820 and PLGA-DEX-IR820/CHT formulations was synthesized and split between the extract assays (Figure 9) and the direct-exposure longitudinal trials (Figure 10). This setup eliminates processing artifacts, confirming that the distinct cellular profiles observed—such as the slight reduction in viability (>70%) for certain extracts compared to the completely benign (>100%) direct-exposure profile—are structurally driven by the nature of the exposure method rather than batch-to-batch anomalies. Across multiple distinct synthesis batches, the platform demonstrated robust reproducibility, exhibiting minor batch-to-batch standard deviations in loading and encapsulation metrics (e.g., EEDEX = 55.9 ± 2.5% and EEIR820 = 40.8 ± 0.5%), well within acceptable pharmaceutical quality standards.

## 4 General Discussion

This work set out to rationalize the design of polymeric nanoparticles for ocular delivery by linking formulation choices to release behavior and, crucially, to spatial transport under ocular-like microflows. Fabrication parameters were systematically varied and read out in terms of drug loading (DL), encapsulation, size, ζ-potential, PDI, coating success, release behavior, photothermal responsiveness, and preliminary cytocompatibility.

Uncoated nanoparticles were consistently small and monodisperse (∼90–100 nm; PDI 0.13–0.19) with high DEX loading/encapsulation (≈7.6%/≈47.6%), which increased upon IR820 coloading (≈8.9%/≈55.9%) without destabilizing size or PDI. Dexamethasone quantification relied on spectrophotometric measurements in complex matrices. While this high-throughput approach was fully validated with clean calibration standards (*R* ^2^ = 0.99) and proved highly reliable for the controlled, protein-free buffer systems used in these initial in vitro screening assays, in principle, this approach may affect baselines due to residual particulates, surfactant micelles, or chitosan, and would benefit from orthogonal analytical confirmation in subsequent biological phases.

Across methods, chitosan coating reliably inverted ζ to positive values (+32 to +41 mV), increased the hydrodynamic diameter to ∼450–500 nm, and was tunable through acid concentration, exposure time, and wash chemistry. Coating at pH ∼ 3.5 while including NaCl in the coating solution proved effective in the nanoprecipitation track. Postprocessing stability in PBS/Tween at 37 °C and after freeze–thaw/reconstitution was preserved within ∼±10%, indicating a robust formulation. It is worth noting that while initial in vitro characterization and release studies were conducted in a lower ionic strength medium (5 mM PBS) to preserve electrostatic stabilization and minimize salt-induced double-layer compression, the translation of these positively charged nanocarriers to physiological environments must consider ionic screening. In the posterior segment of the eye, the vitreous humor is characterized by a dense, viscoelastic network of hyaluronic acid and collagen. This highly macromolecularly crowded physiological matrix introduces significant steric hindrance and structural containment, which typically counteracts classic salt-induced aggregation observed in simplified, surfactant-free 1× PBS solutions. Consequently, the platform’s demonstrated stability in surfactant-supplemented media at 37 °C over extended periods underscores its structural resilience under simulated physiological conditions. While the electrostatic and noncovalent interactions between the cationic primary amines of chitosan and the sialic acid residues of ocular mucin are heavily established in ophthalmic literature as a mechanism for extending residence times, direct ex vivo mucoadhesive tracking was not executed in this primary design stage. Consequently, the prolonged retention benefits of this core–shell framework are currently characterized as a theoretical and literature-supported rationale, which serves as the baseline for our upcoming ex vivo mucosal binding assays.

The architectural expansion from the core matrix to a final core–shell diameter of ∼450−500 nm represents a deliberate, preengineered design choice tailored to optimize localized ocular pharmacokinetics. Rather than aiming for high systemic or trans-vitreal clearance, this platform was specifically developed to act as an in situ long-acting therapeutic depot upon intravitreal administration. The increased steric volume, coupled with the dense hydrophilic chitosan shell, serves a dual purpose: it restricts rapid, nonspecific trans-vitreal migration that leads to early clearance, while simultaneously leveraging mucoadhesive interactions to anchor nanoparticles in place. This immobilization minimizes rapid elimination through anterior outflow tracks, ensuring a highly durable and localized drug reservoir in the vitreous.

Release behavior proved formulation dependent, reaching ∼18% (uncoated) and ∼8% (coated) of the total loaded drug by 336 h. Early time fitting favored Korsmeyer–Peppas for uncoated particles (*R* ^2^ ≈ 0.95; *n* ≈ 0.47, anomalous transport) and Higuchi for coated particles (*R* ^2^ ≈ 0.99), supporting a picture in which the chitosan shell adds an effective diffusional barrier.

The light-triggered release protocol applied to the PNPs revealed a clear early phase enhancement of DEX release (≈2.0–2.3× vs nonirradiated intervals) that diminished as the system approached late-phase equilibrium, indicating practical on-demand dose modulation in the clinically relevant early window. The observed 2.0–2.3-fold acceleration in dexamethasone release kinetics during active NIR laser irradiation cycles is directly driven by photothermal phase behaviors within the polymer matrix. The glass transition temperature (*T*_g_) of standard uncapped PLGA formulations typically ranges between 42 and 48 °C. Our real-time thermal monitoring shows that the encapsulated IR820 dye generates a localized thermal spike of Δ*T* ≈ 7.6 °C, elevating the immediate particulate microenvironment from the baseline ocular temperature (∼34.5 °C) to a peak of ∼42.1 °C. This localized heat hits the lower onset boundary of the PLGA core’s *T*_g_, triggering a rapid ’glass-to-rubber’ state transition. The resulting increase in free volume and polymer chain flexibility dramatically elevates the diffusion coefficient of the encapsulated dexamethasone, allowing it to rapidly escape through the porous networks during the active laser windows.

Cytocompatibility was acceptable within the tested windows. Extract assays were largely benign (viability comparable to negative controls), with only slight reductions (>70%) observed for two coated emulsion formulations. Direct-exposure assays using the final light-responsive formulations (±chitosan) at 25–100 μg mL^–1^ for 24/72 h showed no loss of viability, in line with the established biocompatibility of PLGA, chitosan, and IR820. The selection of the NIH-3T3 mouse fibroblast cell line for the primary cytocompatibility screenings was based on standardized toxicological frameworks. In accordance with ISO 10993-5 protocols for the biological evaluation of medical devices and biomaterials, established fibroblast lines serve as highly sensitive, robust, and universally benchmarked models for mapping out initial extract-induced cytotoxicity and material-driven apoptotic profiles. While generic lines are instrumental for this baseline safety clearance phase, transitioning to specialized ocular lineages—such as retinal pigment epithelial cells (ARPE-19)—represents the next developmental milestone to evaluate tissue-specific interactions during functional efficacy profiling.

From these data, a set of practical design rules emerges. ACN/DMF-based nanoprecipitation represents an ideal method when small, narrow-PDI cores with high loading/encapsulation and slower baseline release are required. ACN:DMF (9:1) supports coloading of Dexamethasone and IR820 without destabilizing colloids. For coating, targeting ζ ≈ +30–40 mV via exposure to NaCl-enriched pH ∼ 3.5 chitosan solutions provides stable shells while protecting drug loading and yielding sustained drug release profiles.

The final parameters chosen for the nanoprecipitation track—including polymer concentrations (20 mg/mL in the stock organic phase) and a 9:1 v/v ACN:DMF cosolvent ratio—were established based on preliminary optimization screenings. These screening phases evaluated the impact of varying polymer-to-drug ratios on colloidal stability, ensuring maximized encapsulation efficiency (47.6 ± 3.3%) while maintaining tight, monodisperse nanoscale core dimensions (∼100 nm) suitable for ocular delivery. Regarding the photothermal component, the physical loading concentration of the NIR dye IR820 was optimized to support robust thermal activation. Under the evaluated dosing regimen, the incorporated dye percentage was sufficient to mediate a reproducible 7.6 °C temperature spike across multiple sequential laser cycles. The observed localized temperature elevation falls safely within the established biocompatibility thresholds for intraocular applications. Because the physiological baseline temperature of the human vitreous and posterior segment rests at ∼34.5 °C—owing to continuous anterior thermal dissipation—this laser-induced spike elevates the immediate peri-particulate microenvironment to a maximum peak of ∼42.1 °C. This remains strictly below the critical threshold for thermal tissue denaturation or retinal protein coagulation. Additionally, due to the microscale thermal mass of the nanoparticle suspensions, this heat generation is highly confined and undergoes rapid, transient dissipation into the surrounding vitreous sink upon laser deactivation, preventing chronic thermal stress. Localized photobleaching of the cyanine chromophore was also observed postactivation; nonetheless, this structural breakdown is restricted to the postirradiation phase, thereby serving as an inherent safety mechanism that limits prolonged, unintended intraocular heat generation.

The present work also highlights several limitations and delineates clear near-term priorities for the here-reported research. Dexamethasone quantification relied on spectrophotometric measurements at 255 nm in complex matrices. In principle, this approach may affect baselines due to residual particulates, surfactant micelles, or chitosan, and would benefit from orthogonal analytical confirmation. In addition, while the functional coating improved nanoparticle performance, the underlying mechanism—whether driven by surface localization or barrier effects—remains indirectly inferred, in the absence of direct visualization or surface-chemical characterization. Cytocompatibility results are encouraging, and they can be broadened in scope by including other cell lines and further functional end points besides cell viability. We also recognize that the simplified, cell-free in vitro environments utilized in this work do not fully replicate the specialized anatomical and physiological transport barriers of the posterior eye. While establishing the fundamental core–shell engineering, charge inversion, and light-triggered release kinetics requires these isolated, baseline setups, evaluating the formulation’s performance in specialized ex vivo trans-vitreal diffusion models or multilayered trans-epithelial electrical resistance (TEER) barrier assays represents a critical and necessary phase for our future preclinical validation tracks. Finally, process robustness and critical quality attributes, including batch-to-batch variability and residual impurities, deserve to be systematically addressed.

Accordingly, near-term efforts should focus on strengthening the mechanistic understanding while expanding biological and translational relevance. Confirmatory quantification methods and matrix-matched calibration strategies would improve mass-balance confidence and data robustness. Elucidating the coating mechanism through complementary imaging and surface-analysis approaches would clarify structure–function relationships, while extended electrokinetic characterization would help in contextualizing performance across physiological conditions. Further formulation stress testing and stability assessment in physiologically relevant media are warranted, alongside a more comprehensive evaluation of photothermal performance and safety under repeated activation. Finally, broadening biological validation to include relevant ocular cell types and barrier models, together with systematic process optimization and quality assessment, will be essential to support future translational development.

Therefore, the next phase of this work will focus on consolidating the experimental pipeline into a predictive framework for ocular drug delivery. Priority will be given to refining analytical accuracy (HPLC validation, mass-balance closure), visualizing coating architecture (TEM, XPS), and extending biological relevance through RPE and endothelial cocultures.

While the present work establishes the baseline colloidal and photothermal stability of the dual-functional nanocarriers within controlled, synthetic PBS environments, we acknowledge that these simplified systems do not fully replicate the macromolecular complexity of real biological matrices. Evaluating matrix stability and structural retention in more complex environments—such as vitreous humor models or protein-rich cell culture media—represents a vital next step for translational development to thoroughly map out potential opsonization or soft-corona interactions prior to in vivo deployment.

Overall, this work delivers a cohesive framework for ocular nanoparticle delivery that spans the formulation, kinetics, and transport. On the materials side, it establishes reproducible PLGA systems with chitosan and IR820, achieving controlled sizes, quantified loading/encapsulation, and documented release profiles. By implementing feasible and concrete next steps, the platform will move from descriptive to decisive, capable of rationally steering light-tunable, mucoadhesive nanomedicine designs for the posterior eye.

## Supplementary Material



## Data Availability

The data supporting the findings of this study are available within the article.

## Notes

Table S1: DLS-Z results for all formulations; Table S2: UV/vis (Nanodrop) results for all loaded formulations in terms of loading efficiency (LE %) and encapsulation efficiency (EE %); Figure S1: Drug release profile for PLGA-DEX and PLGA-DEX/CHT NPs; Table S3: Release modeling results for PLGA-DEX and PLGA-DEX/CHT formulations in terms of goodness of fit (*R* ^2^) and characteristic parameters for various models; Figure S2: Modeled vs raw data for the two best fits for PLGA-DEX and PLGA-DEX/CHT formulations, respectively, Korsmeyer/Peppas (K/P) and Higuchi, and the graphs display the first 5 time points; and Figure S3: visual proof of sample bleaching following three cycles of 800 nm NIR irradiation at an average irradiance of 310 mW/cm^2^(PDF)
