# Supplementary material for: Light-Triggered and Sustained Delivery of Dexamethasone Using Chitosan-Coated PLGA Nanoparticles for Posterior Eye Disease Treatment
Source: ACS Omega. 2026 Jun 26;11(27):40218–31. doi: 10.1021/acsomega.6c02332 (PMC13382836; doi:10.1021/acsomega.6c02332)
Supplement: Supplementary file 1 [file ao6c02332_si_001.pdf]

# Light-Triggered and Sustained Delivery of Dexamethasone Using Chitosan-Coated PLGA Nanoparticles for Posterior Eye Disease Treatment

# SUPPLEMENTARY MATERIAL

Lorenzo Guidi[a*], Maria Grazia Cascone[a], Gaia Riccio[a], Sofia Patri[b], Rupali Dabas[b] Lorenzo Lavista[c], Andrea Camposeo[c], Dario Pisignano[c], Elisabetta Rosellini[a], Nazila Kamaly[b*]

[a]*Department of Civil and Industrial Engineering, University of Pisa, Pisa, Italy*
[b]*Department of Chemistry, Imperial College London, London, United Kingdom*
[c] *Istituto NanoscienzeCNR, Pisa, Italy*

****Corresponding authors**: nazila.kamaly@imperial.ac.uk; lorenzo.guidi@phd.unipi.it*

## 1. SUPPLEMENTARY MATERIAL

*Table S1: DLS-Z results for all formulations*

| Formulation | Z-average | PDI | ζ-potential |
|---|---|---|---|
| **PLGA NPs** | 90.9 ± 1.8 nm | 0.13 ± 0.02 | -12.8 ± 2.6 mV |
| **PLGA/CHT NPs** | 488.4 ± 20.9 nm | 0.4 ± 0.01 | +41.2 ± 2.2 mV |
| **PLGA-DEX NPs** | 96.5 ± 0.6 nm | 0.17 ± 0.02 | -22.4 ± 4.6 mV |
| **PLGA-DEX/CHT NPs** | 456.7 ± 9.8 nm | 0.23 ± 0.02 | +32 ± 6.3 mV |
| **PLGA-DEX-IR820 NPs** | 100.1 ± 5.7 nm | 0.19 ± 0.04 | -12.9 ± 1.7 mV |
| **PLGA-DEX-IR820/CHT NPs** | 496.2 ± 14.4 nm | 0.24 ± 0.04 | +40.7 ± 1 mV |

*Table S2: UV/Vis (Nanodrop) results for all loaded formulations in terms of Loading efficiency (LE %) and Encapsulation efficiency (EE %)*

| Formulation | LE% DEX | EE% DEX | LE% IR820 | EE% IR820 |
|---|---|---|---|---|
| **PLGA-DEX NPs** | 7.6 ± 0.5 % | 47.6 ± 3.3 % | / | / |
| **PLGA-DEX/CHT NPs** | 7.1 ± 1.7 % | 44.1 ± 10.6 % | / | / |
| **PLGA-DEX-IR820 NPs** | 8.9 ± 0.4 % | 55.9 ± 2.5 % | 8.11 ± 0.09 % | 45.9 ± 0.5 % |
| **PLGA-DEX-IR820/CHT NPs** | 7.9 ± 0.4 % | 49.5 ± 2.7 % | 7.3 ± 0.4 % | 40.8 ± 0.5 % |

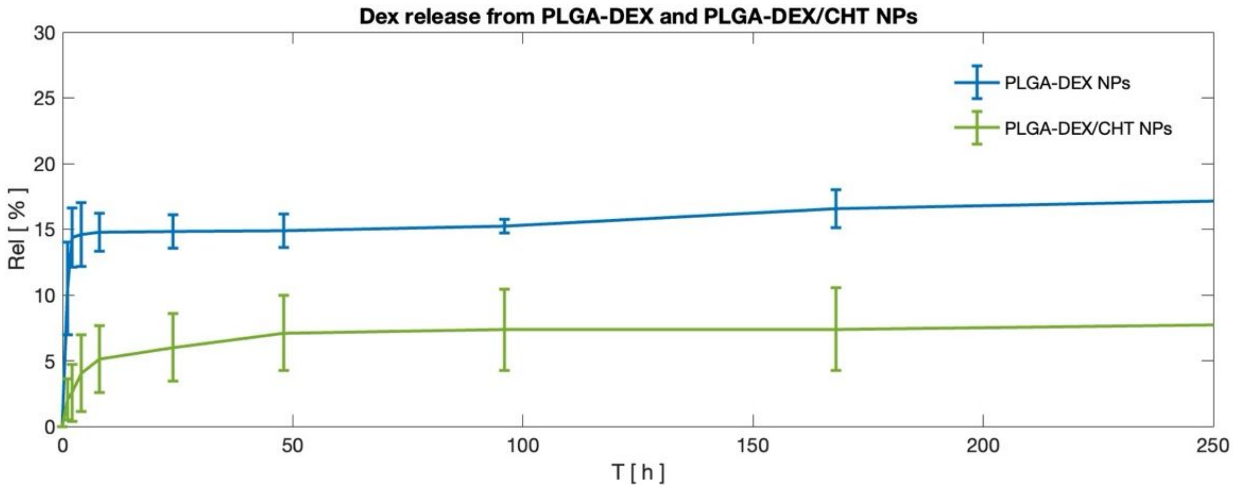


*Figure S1: Drug release profile for PLGA-DEX and PLGA-DEX/CHT NPs.*

*Table S3: Release modeling results for PLGA-DEX and PLGA-DEX/CHT formulations in terms of goodness of fit (R2) and characteristic parameters for various models.*

| Fitting model | PLGA-DEX NPs | PLGA-DEX/CHT NPs |
|---|---|---|
| **Korsmeyer-Peppas** | $\boldsymbol{R^2 = 0.95}$<br>$n = 0.47$<br>$K_p = 1.16$ | $R^2 = 0.92$<br>$n = 0.25$<br>$K_p = 0.91$ |
| **Higuchi** | $R^2 = 0.75$<br>$K_H = 0.66$ | $\boldsymbol{R^2 = 0.99}$<br>$K_H = 0.24$ |
| **Hixson-Crowell** | $R^2 = 0.45$<br>$K_{HC} = 3.62 * 10^{-4}$ | $R^2 = 0.86$<br>$K_{HC} = 1.51 * 10^{-4}$ |
| **Zero-order** | $R^2 = 0.44$<br>$K_0 = 0.02$ | $R^2 = 0.86$<br>$K_0 = 0.01$ |
| **First-order** | $R^2 = 0.45$<br>$K_p = -2.41 * 10^{-4}$ | $R^2 = 0.86$<br>$K_p = -9.86 * 10^{-4}$ |

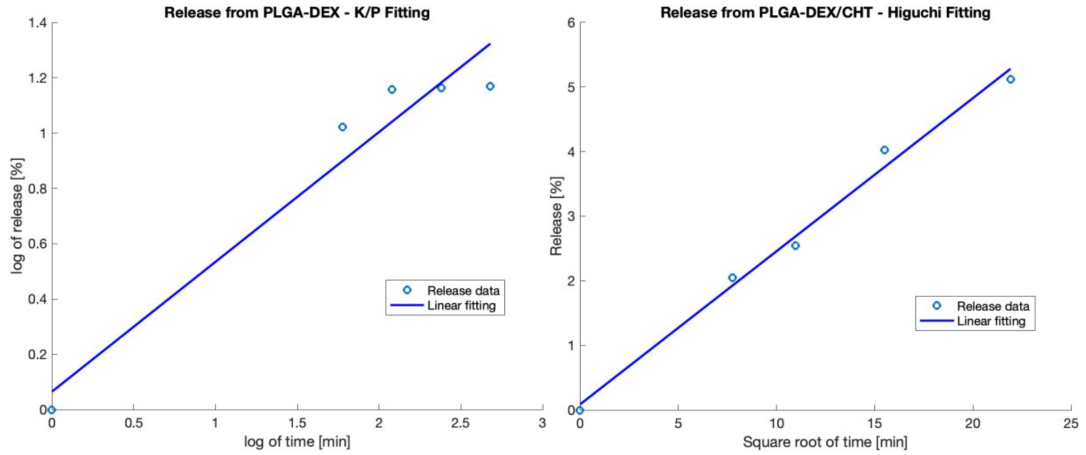


*Figure S2: Modelled vs raw data for the two best fits for PLGA-DEX and PLGA-DEX/CHT formulations, respectively Korsmeyer/Peppas (K/P) and Higuchi. The graphs display the first 5 timepoints.*

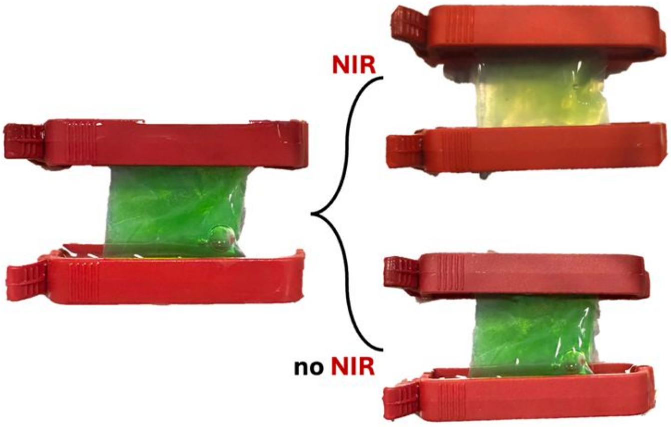


*Figure S3: Visual proof of sample bleaching following three cycles of 800nm NIR irradiation at an average irradiance of 310 mW/cm².*
